# A nomogram to predict ventricular thrombus in dilated cardiomyopathy patients

**DOI:** 10.1007/s11239-023-02846-2

**Published:** 2023-06-23

**Authors:** Xiao-Lei Li, Dilare Adi, Yun Wu, Aibibanmu Aizezi, Yan-Peng Li, Munawar Kerem, Xian Wei, Fen liu, Xiang Ma, Yi-Tong Ma

**Affiliations:** 1 State Key Laboratory of Pathogenesis, Prevention and Treatment of High Incidence Diseases in Central Asia, Department of Cardiology, First Affiliated Hospital of Xinjiang Medical University, Urumqi, 830054 Xinjiang China; 2 Xinjiang Key Laboratory of Cardiovascular Disease, Clinical Medical Research Institute, First Affiliated Hospital of Xinjiang Medical University, Urumqi, 830054 Xinjiang China; 3 State Key Laboratory of Pathogenesis, Prevention and Treatment of High Incidence Diseases in Central Asia, Department of General Medicine, First Affiliated Hospital of Xinjiang Medical University, Urumqi, 830011 China

**Keywords:** Dilated cardiomyopathy, Ventricular Thrombus, Embolism, Nomogram

## Abstract

Background: VT (Ventricular Thrombus) is a serious complication of dilated cardiomyopathy (DCM). Our goal is to develop a nomogram for personalized prediction of incident VT in DCM patients. Methods: 1267 patients (52.87 ± 11.75 years old, 73.8% male) were analyzed retrospectively from January 01, 2015, to December 31, 2020. A nomogram model for VT risk assessment was established using minimum absolute contraction and selection operator (LASSO) and multivariate logistic regression analysis, and its effectiveness was validated by internal guidance. The model was evaluated by the area under the receiver operating characteristic curve (AUC), calibration curves, and decision curve analysis (DCA). We compared the performance in predicting VT between nomogram and CHA2DS2, CHA2DS2- VASc or ATRIA by AUC, akaike information criterion (AIC), bayesian information criterion (BIC), net reclassification index (NRI), and integrated discrimination index (IDI). Results: 89 patients (7.02%) experienced VT. Multivariate logistic regression analysis revealed that age, left ventricular ejection fraction (LVEF), uric acid (UA), N-terminal precursor B-type diuretic peptide (NT-proBNP), and D-dimer (DD) were important independent predictors of VT. The nomogram model correctly separates patients with and without VT, with an optimistic C score of 0.92 (95%CI: 0.90–0.94) and good calibration (Hosmer-Lemeshow χ^2^ = 11.51, *P* = 0.12). Our model showed improved prediction of VT compared to CHA2DS2, CHA2DS2-VASc or ATRIA (all *P* < 0.05). Conclusions: The novel nomogram demonstrated better than presenting scores and showed an improvement in predicting VT in DCM patients.

**Supplementary Information:**

The online version contains supplementary material available at 10.1007/s11239-023-02846-2.

## Introduction

Ventricular thrombus (VT) was a dreaded complication in patients with severe ventricular dysfunction [1]. Studies had confirmed VT have a higher risk of cerebral, peripheral arterial, systemic embolism, and long-term mortality [2]. Despite innovations in medical technology and optimization of anticoagulant solutions, the current situation in VT is not promising.

Patients with DCM were at risk of thrombosis/embolism due to increased heart size, decreased ventricular wall motion, and blood flow arrest [3, 4]. Previous studies have shown that DCM is most likely to develop cardiac cavity thrombosis, with an estimated incidence of 4–44% [5, 6] in DCM patients. Furthermore, 12.8% of patients with cardiogenic stroke were caused by DCM thrombosis [7]. A study that analyzed the results of 11,724 autopsies also showed that about 2.4% of patients had undetected intracardiac thrombosis [8]. Studies have shown that patients with VT (VT) have poor clinical outcomes, but reasonable anticoagulation therapy could effectively abate VT formation and thus mortality [9]. Unfortunately, DCM lacks effective tools for early identification of VT risks, and current anticoagulation protocols regimens are mostly based on clinical experience and expert consensus [10, 11]. Few large-scale clinical studies had focused on diagnosing, treating and preventing VT in this specific population of DCM. To our knowledge, there are no available predictive models to predict the occurrence of DCM thrombosis.

In our study, we aimed to identify risk factors for VT in DCM patients and develop a predictive nomogram model to evaluate the risk of VT in individual patients.

## Materials and methods

### Study Population

Our study was a part of clinical research which was named Clinical Characteristics and Prognosis of Patients with Primary Cardiomyopathy in Xinjiang. This was a retrospective cohort study conducted by the Department of Cardiology, First Affiliated Hospital of Xinjiang Medical University. Design details are available at www.chictr.org.cn (Registration number: ChiCTR2200058051). The aim of the study was evaluating the clinical features and prognostic factors of cardiomyopathy.

**Fig. 1:**
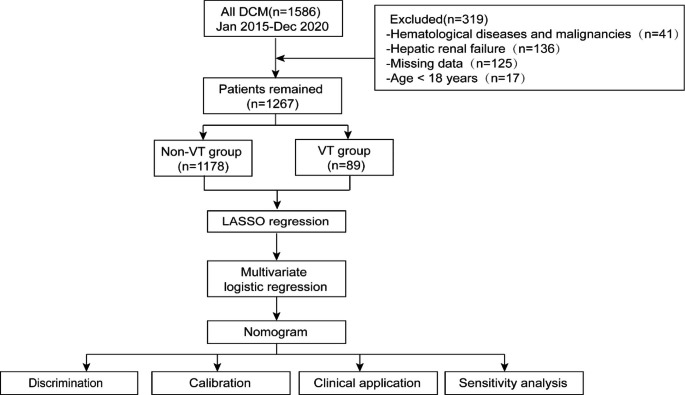
Flow diagram of the study. VT, ventricular thrombus

A total of 1586 patients with DCM who attended to the First Affiliated Hospital of Xinjiang Medical University were collected from January 01, 2015, to December 31, 2020. After initial evaluation 319 were excluded, ultimately a total of 1267 patients were included into this study of which 90 with VT (VT group) and 1180 without VT (non-VT group). The case screening and study flow was shown in Fig. 1. The inclusion criteria were as follows [11]: (1) left ventricular end-diastolic dimension (LVEDd) > 5.5 cm in male and LVEDd > 5.0 cm in female; (2) left ventricular fractional shortening < 25% and/or left ventricular ejection fraction (LVEF) < 45%. We excluded patients who (1) patients with ischemic heart disease, hypertensive heart disease, valvular heart disease or congenital heart disease; (2) combined with severe hepatic and renal insufficiency, hematological system diseases, malignant tumors; (3) patients younger than 18 years of age; (4) those with incomplete clinical data.

### Research Content

Information on patient demographics, tests/examinations, major comorbidities, medications, and intracardiac thrombus distribution were collected retrospectively. All data were obtained for their first measurement at admission. In addition, the CHADS2 score, CHA2DS2VASc score, and ATRIA score were calculated for each patient based on previously published criteria.

The CHADS2 was counted by giving 1 point for each factor such as heart failure, hypertension, age ≥ 75 years and diabetes mellitus, and 2 points for a history of transient ischemic attack (TIA) and/or stroke [12]. The CHA2DS2-VASc score was the total of points after the addition of one point each for heart failure, hypertension, diabetes, vascular disease, age 65–74 years, and female sex and two points each for previous stroke or TIA and age ≥ 75 years [13]. The criteria for ATRIA score refer to table S1 [14].

### Statistical analysis

All analyses were performed using Social Package for the Social Sciences (SPSS) version 26.0 (SPSS Inc., Chicago, IL, USA), and R software version 4.0.3 (https://cran.r-project.org). The R software mainly include “glmnet”, “caret”, “rms”, “pROC”, “rmda”, and so on.

Categorical variables were expressed as counts (percentage, %) and continuous variables were expressed as mean ± standard deviation (SD) or median (25th, 75th percentiles). The differences in baseline characteristics between the two groups were examined by Student t-Test or Mann–Whiney U-test for continuous variables and the Pearson chi-square test (Pearson χ^2^ test) or Fisher exact test for categorical variables, as appropriate. All tests were 2-sided, and *P* value < 0.05 was considered statistically significant.

### Model development and validation

The least absolute shrinkage and selection operator (LASSO) regression was used to screen the non-zero coefficient characteristic variables, all of which were analyzed using multivariate logistic regression analysis. A nomogram model was developed based on multivariate logistic regression results. Nomogram performance was evaluated by both discriminations, presented as C-index and the area under the receiver operating characteristic curve (AUC), and calibration, expressed as the Hosmer-Lemeshow test and calibration plot. Clinical effectiveness was visualized in the decision curve (DCA). Bootstrapping method with 1000 repetitions were used to validate the performance of the nomogram. By comparing the nomogram and CHA2DS2, CHA2DS2-VASc or ATRIA score, the discriminative ability and goodness of fit were valuated with AUC, akaike information criterion (AIC), bayesian information criterion (BIC), net reclassification index (NRI) and integrated discrimination index (IDI).

## Results

### Outcomes

In this study, we retrospectively analyzed 1267 patients diagnosed with DCM (mean age 52.87 ± 11.75, 73.8% male). Among them, 89 (7.08%) of 1267 patients with DCM had combined VT, with unilateral left ventricular predominance (78/89 cases, 87.64%) and biventricular thrombus was rare (4/89 cases, 4.49%). The distributions of thrombus location were left ventricular apical (61/89 cases, 68.54%), middle left ventricle (17/89 cases, 19.10%), right ventricle (7/89 cases, 7.86%), and biventricular (4/89 cases, 4.49%). Statistical analysis showed no difference in in-hospital mortality between the two groups (4.49% vs 3.14%, P = 0.531), but in-hospital new stroke was higher in VT group (7.87% vs 2.71%, P = 0.006), and the mean length of stay was significantly longer in VT group (9.98 vs 8.02 days, P = 0.001). (Table S2)

### Patient characteristics

Compared with the non-VT group, as shown in Table 1, in the VT group, there were older age (*P* = 0.012), higher proportion of males (*P* = 0.0380), higher New York Heart Association class (*P* < 0.001), higher proportion of urine protein (*P* < 0.001), higher ATRIA score (*P* = 0.024), higher levels of N-terminal precursor B-type diuretic peptide (*P* < 0.001), aspartate aminotransferase (*P* = 0.001), alanine transaminase (*P* < 0.001), creatinine (*P* < 0.001), urea (*P* = 0.008), Uric Acid (*P* < 0.001) and DD (*P* < 0.001). Systolic blood pressure (*P* = 0.043) and serum albumin (*P* < 0.001) were significantly lower than those in the VT group. Echocardiographic results also showed that the left atrial (LA), right atrial (RA), and right ventricular (RV) diameters were larger in VT group, while LVEF was significantly lower (all *P* < 0.05). And there was lower proportion of mitral regurgitation in the VT group (*P* = 0.045).

**Table 1:** Baseline characteristics of patients

Variable	Total	Non-VT group	VT group	*P* Value
Age, (years) Gender, (Male, n%) Ethnic, n (%)	54 (46, 61.5) 935 (73.8)	54 (45, 61) 861 (73.1)	57 (47, 65) 74 (83.1)	0.012 0.038
Han	642 (50.7)	602 (51.1)	40 (45.0)	0.402
Uygur	424 (33.4)	393 (33.4)	31 (34.8)	
Other races	201(15.9)	183 (15.5)	183 (15.5)	
Smoke, n (%)	477 (37.6)	442 (37.5)	35 (39.3)	0.735
Drinking, n (%)	289 (22.8)	269 (22.8)	20 (22.5)	0.937
NYHA, n (%)				
Grade I-II	198 (15.6)	191 (16.2)	7 (7.9)	< 0.001
Grade III	748 (59.0)	706 (59.9)	42 (47.2)	
Grade IV	321 (25.4)	281 (23.9)	40 (44.9)	
Weight (kg)	75 (64, 85)	75 (67, 82)	73.5 (65, 83)	0.564
SBP, (mmHg)	116 (105, 127)	117 (105, 127)	110 (100, 130)	0.043
DBP, (mmHg)	75 (67, 83)	75 (67, 82)	75 (65, 83)	0.622
Medical history, n (%)				
Hypertension	368 (29.0)	347 (29.5)	21 (23.6)	0.240
Diabetes mellitus	176 (13.9)	163 (13.8)	13 (14.6)	0.840
Pre-Stroke	38 (3.0)	34 (2.9)	4 (4.5)	0.391
AF	207 (16.3)	198 (16.8)	9 (10.1)	0.099
COPD	94 (7.4)	90 (7.6)	4 (4.5)	0.275
Urineprotein	109 (8.6)	91 (7.7)	18 (20.2)	< 0.001
Laboratory characteristics				
WBC, (109 /L)	7.00 (5.68, 8.50)	7.0 (5.65, 8.49)	7.15 (5.89, 9.43)	0.255
RBC, (109 /L)	4.77 (4.39, 5.15)	4.75 (4.38, 5.14)	4.92 (4.54, 5.20)	0.061
Hb, (g/L)	143 (130, 153)	142 (130, 153)	143 (132, 153)	0.469
PLT, (109 /L) Creatinine, (ummol/L)	208 (167, 255) 80.00 (67.00, 95.00)	209 (168, 256) 79.30 (67.00, 94.00)	199 (153, 246) 90.44 (74.00, 111.00)	0.175 < 0.001
Urea (mmol/L)	6.40 (5.10, 8.07)	6.33 (5.10, 8.00)	7.23 (5.61, 9.30)	0.008
UA (mmol/L)	422.80 (331.51, 524.58)	416.09 (327.31, 511.60)	491.00 (406.20, 609.38)	< 0.001
ALT, (U/L)	28.23 (18.55, 46.56)	27.90 (18.40, 45.33)	36.01 (20.20, 85.74)	0.001
AST, (U/L)	26.40 (19.80, 36.95)	25.85 (19.60, 35.97)	30.33 (23.20, 58.60)	< 0.001
Albumin, (g/L)	37.70 (34.00, 41.05)	37.92 (34.30, 41.20)	34.44 (31.16, 37.00)	< 0.001
DD/100, (ng/mL)	2.90(1.37, 6.52)	2.63 (1.31, 5.67)	14.04 (10.05, 25.92)	< 0.001
NT-proBNP/100, (ng/mL)	29.97 (12.96, 60.60)	28.37 (12.50, 55.38)	75.67 (40.48, 87.23)	< 0.001
Fibrinogen (g/L)	3.54 (3.07, 4.07)	3.53 (3.07, 4.05)	3.61 (3.13, 4.36)	0.127
Echocardiography characteristics				
LA, (mm)	45 (41, 49)	45 (41, 49)	46 (43, 50)	0.009
LVEDd, (mm)	68 (63, 74)	68 (63, 74)	70 (64, 75)	0.274
RA, (mm)	40 (35, 46)	40 (35, 46)	45 (40, 50)	< 0.001
RV, (mm)	21 (19, 25)	21 (19, 25)	24 (21, 27)	< 0.001
LVEF, (%)	34.00 (30.00, 38.00)	35.00 (30.00, 38.00)	31.00 (25.00, 36.00)	< 0.001
Mitral regurgitation, n (%)	359 (28.3)	342 (29.0)	17 (19.1)	0.045
CHADS2	1 (1, 2)	1 (1, 2)	1 (1, 2)	0.284
CHA2DS2-VASc	1 (1, 2)	2 (1, 2)	2 (1, 3)	0.782
ATRIA	1 (1, 3)	2 (1, 3)	2 (1, 4)	0.024

VT, ventricular thrombus; NYHA, New York Heart Association; SBP, systolic blood pressure; DBP, diastolic blood pressure; AF, atrial fibrillation; COPD, chronic obstructive pulmonary disease; WBC, white blood cell count; WBC, red blood cell count; PLT, platelet count; Hb, hemoglobin; AST, aspartate aminotransferase; ALT, alanine transaminase; UA, Uric Acid; DD,D-dimer; NT-proBNP, N-terminal; precursor B-type diuretic peptide; LA, left atrial diameter; LVEDD, left ventricular end-diastolic diameter; RV, right ventricular diameter; RA, right atrial diameter; LVEF, left ventricular ejection fraction; ACEI, angiotensin converting enzyme inhibitor; ARB, angiotensin receptor blockers; MRA, mineralocorticoid receptor antagonist; NOAC, novel oral anticoagulants; CRT, cardiac resynchronization therapy (CRT); CRTD, cardiac resynchronization therapy defibrillator; ICD, implantable cardioverter-defibrillator

### Variable screening

**Fig. 2:**
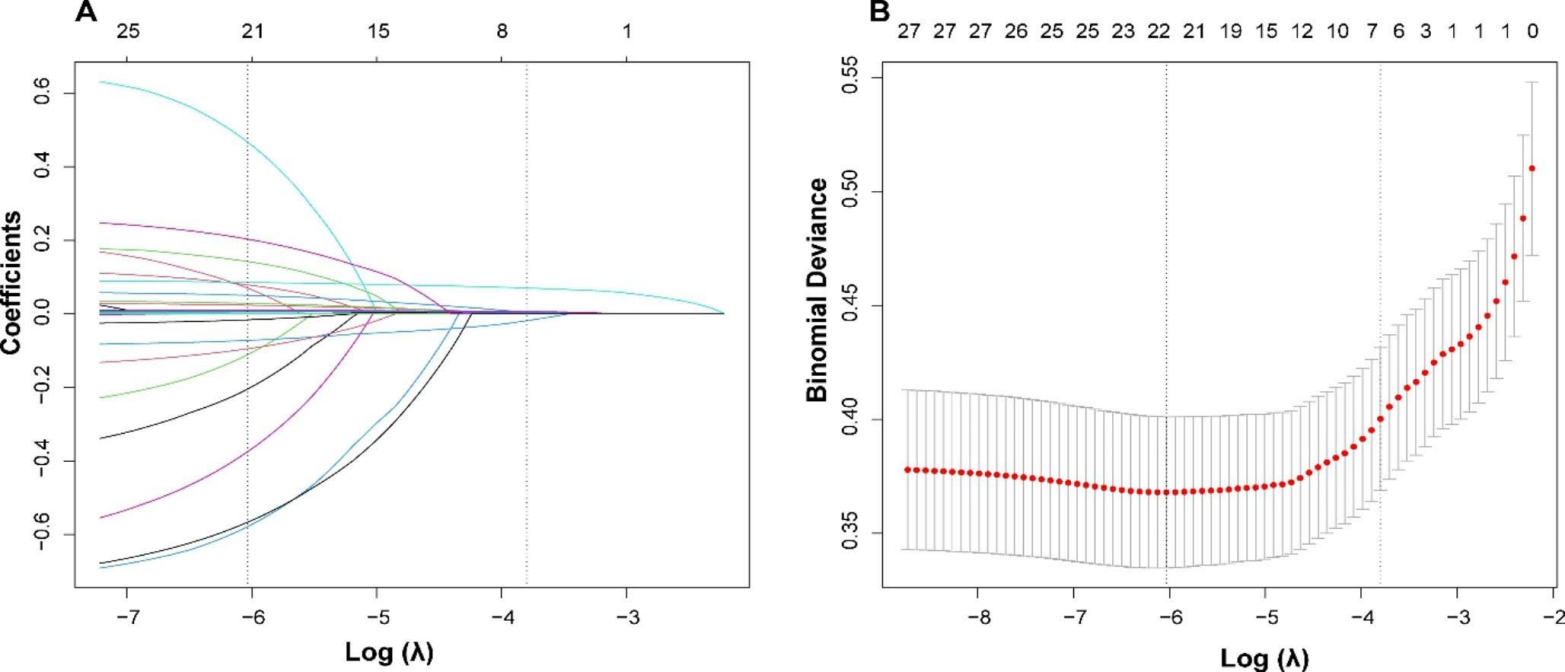
Significant variables selection using the LASSO. A, plot of each variables coefficient profile against log (lambda). B, ten-fold cross-validation used to validate the optimal lambda in the LASSO model. LASSO, the least absolute shrinkage and selection operator (LASSO)

The pre-specified variables were selected based on clinical experience and expert consensus on on thrombosis treatment [10]. Variables considered were smoking, alcohol consumption, AF, pre-stroke, hypertension, diabetes mellitus, urine protein, fibrinogen, mitral regurgitation and LVEDd. In order to select variables that could predict the primary outcome, we chose both pre-specified and statistically significant variables between the VT and non-VT groups, and then entered the LASSO regression analysis. The non-zero coefficient characteristic variables corresponding to the maximum λ within one standard deviation of the mean error is the final model predictor variables. Finally, we have selected seven statistically significant variables including age, LVEF, AST, Crea, UA, NT-proBNP, and DD (Fig. 2). Then in order to simplify the model and make it easier to use, based on optimal cutoff values, we converted these seven possible continuous predictive variables into sub-typed variables. The classification is provided in table S3.

### Model development

Using logistic regression analysis, we reanalyzed the occurrence of VT in patients with DCM, we found that the age [odds ratio (OR): 2.14, 95%confidence interval (CI): 1.26–3.62, *P* = 0.005], LVEF (OR: 2.73; 95% CI: 1.61–4.63; *P* < 0.001), UA (OR: 2.15; 95% CI: 1.28–3.61; *P* = 0.004), NT-proBNP (OR: 2.33; 95% CI: 1.37–3.95; *P* = 0.002) and DD (OR: 31.20; 95% CI: 14.60–66.70; *P* < 0.001) were independent risk factors for unplanned ICU admission in patients with DCM. (Table 2)

**Table 2:** Logistic regression analysis for the occurrence of VT

Variables	Univariate analysis			Multivariable analysis		
	OR	95% CI	P Value	OR	95% CI	*P* Value
Age	1.81	1.17–2.80	0.007	2.14	1.26–3.62	0.005
LVEF	3.09	1.98–4.85	< 0.001	2.73	1.61–4.63	< 0.001
UA	3.17	2.03–4.97	< 0.001	2.15	1.28–3.61	0.004
AST	3.51	2.19–5.63	< 0.001			
Creatinine	2.44	1.56–3.83	< 0.001			
NT-proBNP	6.39	4.04–10.13	< 0.001	2.33	1.37–3.95	0.002
DD	41.95	20.01–87.99	< 0.001	31.20	14.60–66.70	< 0.001

LVEF, left ventricular ejection fraction; UA, Uric Acid; NT-proBNP, N-terminal precursor B-type diuretic peptide; DD, D-dimer; AUC, the area under the receiver operating characteristic curve; OR, odds ratio; CI, confifidence interval

### Nomogram model display

**Fig. 3:**
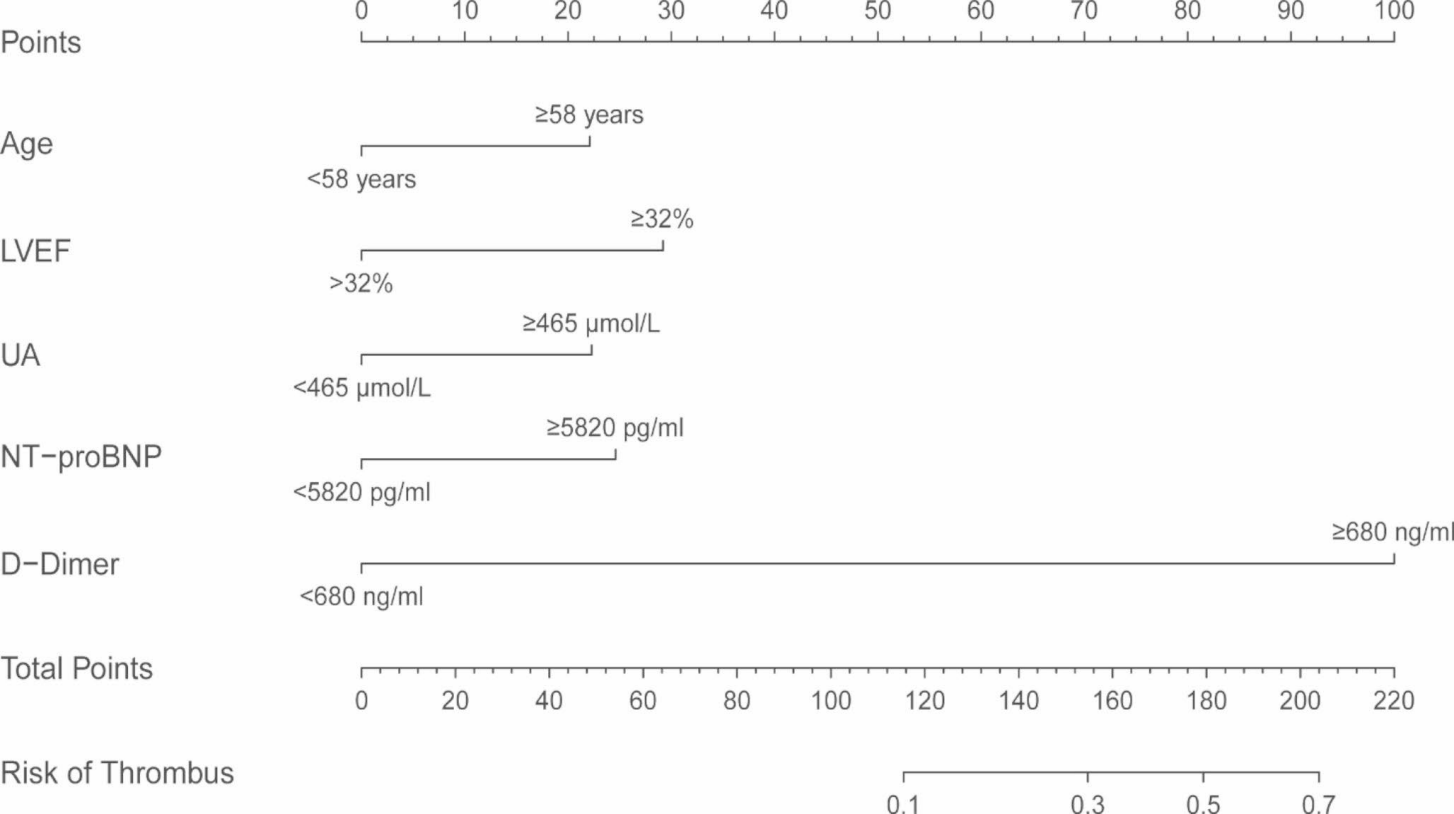
A nomogram to predict the probability of VT of DCM. Points were assigned for each variable by drawing a line upward from the corresponding values to the “points line”. The “total points” was calculated as the sum of the individual score of each of the 5 variables included in the nomogram. We can estimate the risk of VT for this patient by the probability corresponding to the “total points”. UA, Uric Acid; LVEF, left ventricular ejection fraction; NT-proBNP, N-terminal precursor B-type diuretic peptide

Five independent risk variables were used to build a nomogram for predicting the risk of VT in patients with DCM. The scores corresponding to each predictor variable in the nomogram are summed, and the resulting probability value corresponding to the total score is the probability of risk of VT. (Fig. 3)

### Nomogram evaluation and validation

**Fig. 4:**
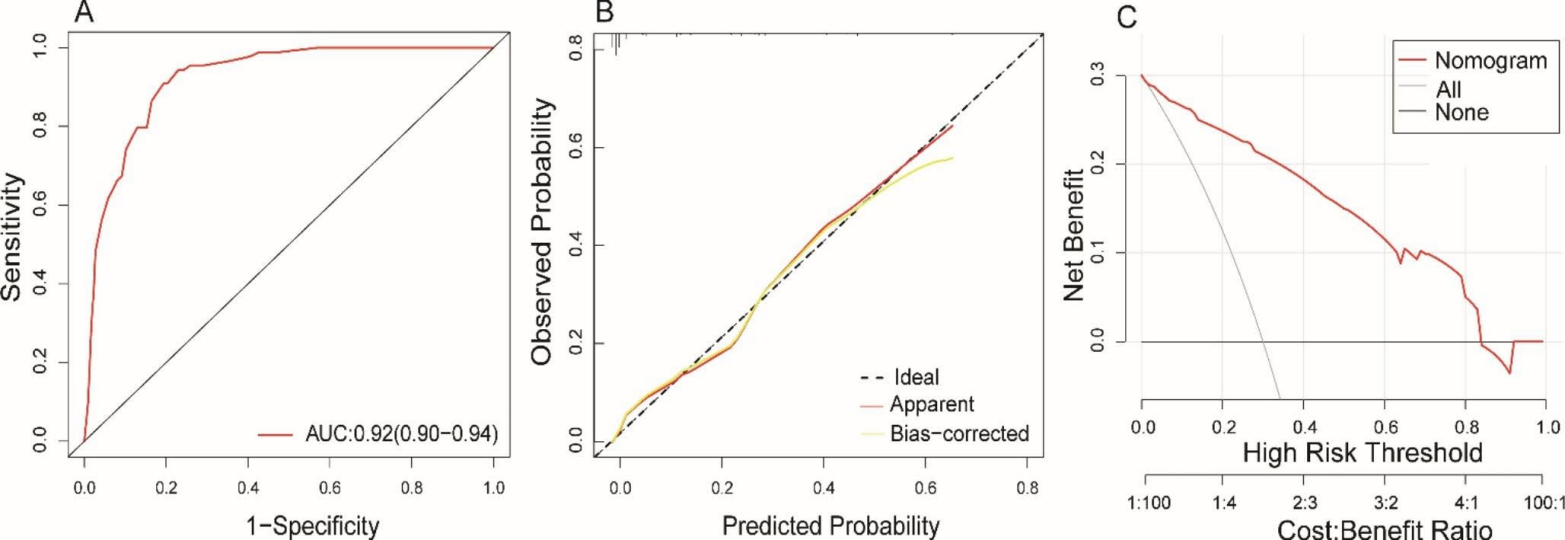
The evaluation and internal validation for nomogram. A, the AUC of nomogram in predicting VT of DCM; B, the calibration curve of the nomogram; C, the decision curve analysis of the nomogram

Our model was highly discriminating with a C index of 0.92 (95% CI: 0.90–0.94) and the AUC of 0.92 (95% CI: 0.90–0.94), as shown in Fig. 4A. The performance of the model was verified by the Bootstrapping method and a good C-index of 0.92 was obtained. To assess calibration ability, the Hosmer-Lemeshow test were performed, and the results showed that the model was calibration ability (χ^2^ = 11.51, *P* = 0.12). Also, the calibration curve shows that the predicted probability curve of the model was very close to the ideal curve, suggesting that the predicted probability of the model for VT was in good agreement with the actual probability of occurrence, as shown in Fig. 4B. Except that, the DCA analysis showed that when the threshold probability of VT was between 0 and 0.83, using nomogram to make clinical decisions resulted in greater net benefit than the “no intervention” or “all intervention” scenarios. Within a reasonable range of threshold probabilities, the predictive model as a whole also yielded higher net benefits than each factor alone(Fig. 4C).

### Model improvement capabilities

**Fig. 5:**
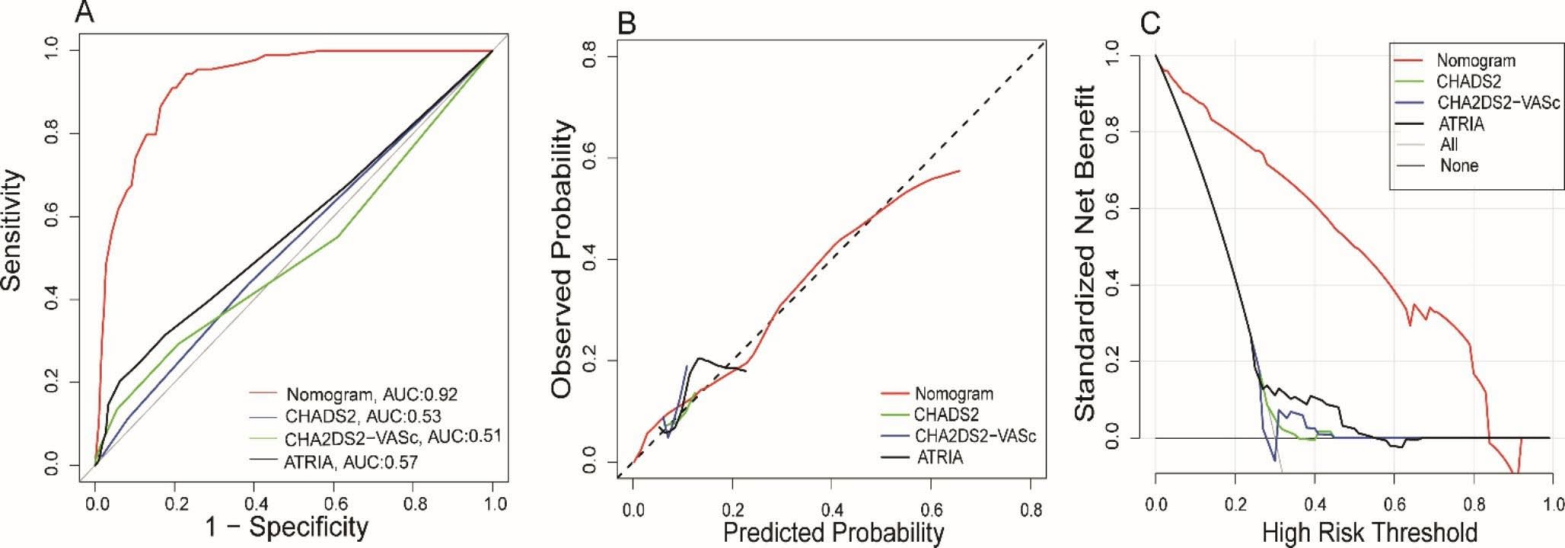
The improvements of the nomogram compared with three scores. Model. A, ROC curve comparing the nomogram and three scores; B, Calibration curve comparing the nomogram and three scores; C, Decision curve comparing the nomogram and three scores

AUC, AIC and BIC were used to examine the discriminative and goodness of fit ability of our nomogram, CHA2DS2, CHA2DS2VASc and ATRIA. As shown in Fig. 5A and Table 3, ROC curve analysis showed that our model performed best. The AUC for our nomogram, CHA2DS2, CHA2DS2VASc, or ATRIA was 0.92 (95% CI: 0.90–0.94), 0.53 (95% CI: 0.47–0.58), 0.51 (95% CI: 0.44–0.57), and 0.57 (95% CI. 0.50–0.63), respectively. The DeLong test suggested a statistically significant difference between the new model and CHA2DS2, CHA2DS2VASc or ATRIA score in the ability to differentiate patients with VT (All *P* < 0.001), Table 3.

**Table 3:** The AUC, AIC and BIC of different models

Variable	AUC(95%CI)	Sensitivit	Specificity	P value	AIC	BIC
Nomogram	0.92 (0.90–0.94)	91%	81%	reference	411.61	442.48
CHA2DS2	0.53 (0.47–0.58)	44%	61%	< 0.001	646.68	656.97
CHA2DS2VASc	0.51 (0.44–0.57)	29%	79%	< 0.001	646.38	656.67
ATRIA	0.57 (0.50–0.63)	20%	94%	< 0.001	636.02	646.31

AUC, the area under the receiver operating characteristic curve; OR, odds ratio; CI, confidence interval; AIC, akaike information criterion; BIC, bayesian information criterion

The AIC and BIC for our nomogram were 411.61 and 442.48, whereas those for CHA2DS2, CHA2DS2VASc and ATRIA were 646.68 and 659.97, 646.3 and 656.67, 636.02 and 646.31, indicating that the overall ability was improved by our nomogram (Table 3). Besides, the calibration curve demonstrates excellent calibration in our nomogram (Fig. 5B). Compared to CHA2DS2, CHA2DS2VASc and ATRIA, our nomogram shows an increased net clinical benefit depending on the decision curve (Fig. 5C).

Meanwhile, table S4 showed that the NRI for our model compared to the CHA2DS2, CHA2DS2- VASc or ATRIA score was 1.38 (1.23–1.58, *P* < 0.001), 1.41 (1.26–1.55, *P* < 0.001), 1.39 (1.24–1.53, *P* < 0.001), respectively. Besides, our nomogram had improved IDI value compared to CHA2DS2 (0.27, 95% CI 0.24–0.32, *P* < 0.001), CHA2DS2-VASc (0.28, 95% CI 0.24–0.32, *P* < 0.001) and ATRIA (0.27, 95% CI 0.23–0.31, *P* < 0.001).

### Sensitivity analysis

We performed multiple stepwise regression analysis of the 7 variables screened by LASSO regression as continuous-type variables, and the results showed that 6 factors, including age, LVEF, AST, Crea, UA, NT-proBNP, and DD, were independent predictors of VT and constructed the model2.

ROC curves, calibration plot, and clinical decision curves were used to compare the two models, nomogram and model2. And there were no significant differences between the two models(P > 0.05). But the nomogram model we constructed contained fewer variables and was more convenient for clinical use. See fig S1A-C.

## Discussion

To our knowledge, this is the first VT risk prediction nomogram model for patients with DCM. The model provides an efficient and accurate estimation of VT by using easily gathered clinical parameters: age, LVEF, UA, NT-proBNP, and DD.

### Risk factors

Clinically, patients with DCM are at high risk of thromboembolic events, and intraventricular thrombosis was a significant cause of embolic events [15]. Our study showed that the risk of intracardiac thrombosis in patients with DCM was 7.08% (90/1270). Consistent with the results of other previous investigators [16, 17], the present study also showed that intraventricular thrombus in patients with DCM was predominantly distributed in the left ventricle (67/90), with a higher concentration in the apical region. We also found that, DCM with VT had a significantly higher risk of in-hospital stroke and a significantly longer hospital stay compared with patients without VT.

There is no consistently accepted risk classification model for DCM thrombosis, our model provides a better tool for assessing the risk of VT. In our study the newly developed nomogram model includes only five variables but had well calibrated and good discriminatory ability. The five risk variables included in our nomogram were the most important factors associated with VT in DCM patients. Age is known to be an important predictor of thrombotic and stroke events. Many existing available scores, such as CHADS2, CHA2DS2-VASc, and ATRIA scores [12–14],was adopted age used as risk predictors. Our study also suggests that DCM patients with VT were older, and multiple regression analysis suggests that age over 48 was an independent risk factor for VT in DCM. Our findings are again in line with those of Lemître [18].

To date, several serological markers have been implicated in the occurrence and development of VT. Ample evidence confirms that DD is not only good at in predicting left atrial thrombus in patients with atrial fibrillation, but is also more sensitive and accurate in predicting intraventricular thrombus in patients with heart failure [19–22]. In our study, DD levels were significantly elevated in DCM patients with VT and confirmed that DD may be a most important independent biomarker for predicting VT. Other studies showed that patients with high UA levels were often at high risk for venous thromboembolism, systemic disease embolism and left atrial thrombosis [23–25]. UA was the final product of purine metabolism and efficient oxidizer. UA may promote thrombosis by increasing oxidative stress, promoting inflammatory responses, and through association with comorbidities [26]. Similarly, our data remind that UA was a novel risk factor for VT risk of stratification in DCM. It was well known that hyperuricemia is not uncommon in DCM patients, and we need to be aware of the risk of thrombosis in such patients.

In addition, deterioration of cardiac function was also a significant cause of VT [22]. Traditionally, cardiac function was assessed primarily by LVEF and NYHA, while the NT-proBNP was widely used to assess ventricular function as a major marker of ventricular function. Previous studies had confirmed that LVEF was an important risk factor for LVT formation in DCM patients, and low LVEF was closely associated with the incidence of embolic events [27, 28]. In our study, DCM patients with VT tended to have a lower LVEF (31% vs. 35%), and DCM with low LVEF (< 32%) had a higher risk of VT. At the same time, At the same time, data showed higher levels of NYHA and NT-proBNP in VT patients, consistent with the results of other researchers [29].

### The benefits of our nomogram compared to the other scores

The CHADS2, CHA2DS2VASc, and ATRIA scores are the most frequently employed risk scores and have been proven to be very useful in evaluating thrombosis and stroke risk in patients with atrial fibrillation [12–14]. However, these scores were not satisfactory in predicting thrombosis in patients with heart failure. Zhou et al. [22] showed that CHADS2 score and CHA2DS2-VASc score performed not well with small AUC of 0.52 and 0.55 for predicting stroke in patients with heart failure. Some other literatures reported that CHA2DS2-VASc or ATRIA scores had underestimated stroke risk of heart failure patients [22, 30, 31]. Concordant with these reports, in our study, CHADS2, CHA2DS2-VASc and ATRIA had lower AUC (0.53, 0.50, 0.56), and significantly underestimated the thrombotic risk in DCM patients, as shown in Fig. 5B. Thus, the CHADS2, CHA2DS2-VASc and ATRIA scores developed based on the atrial fibrillation population would not be suitable for DCM patients. Consequently, a new nomogram model developed for specific patients with DCM undergoing VT risk is desperately necessary.

In our study, we developed a prediction nomogram model that showed excellent discrimination with an AUC of 0.92 and was significantly higher than former three scores. In addition, the column line graph showed better calibration and clinical benefit compared to CHADS2, CHA2DS2-VASc and ATRIA scores. We consider that it might be explained by the following reasons: First, our nomogram model was developed specifically for patients with DCM and not just for patients with AF. It is more targeted. Second, our nomogram model was constructed basing on the from clinical characteristics, laboratory tests, and echocardiographic findings, while other scores were developed only upon clinical characteristics. Several clinical risk factors identified from previous scores (stroke, sex, hypertension, and diabetes) were not considered in our nomogram because no independent association with VT was demonstrated. These factors did not have additional prognostic value in the prediction of risk for VT in DCM patients. Third, adequately affirming the role of cardio-functional factors and thrombotic markers may be critical to the accurate prediction of thrombotic events with our model. As in many other studies, DD and LVEF were important indicators of VT in patients with DCM [22, 27].

Our study also has some limitations. First, retrospective and selective biases were inevitable due to single-center, retrospective, small-sample studies. Therefore, con-ducting large-sample, multicenter, prospective studies is still necessary to improve the accuracy and applicability of the model. Second, although this study internally validated the model, it did not receive other external independent cohorts for validation. Therefore, the clinical usefulness of this prediction model needs to be evaluated and validated in other large patient cohorts to improve its calibration capabilities. This will be an important element of our future work.

## Conclusion

In summary, we developed a nomogram model to predict the risk of VT in patients with DCM. Compared to CHADS2, CHA2DS2VASc and ATRIA scores, the novel nomogram model is highly discriminative and clinically effective.

### Electronic supplementary material

Below is the link to the electronic supplementary material.

Supplementary Material 1

## Data Availability

Data sets used and/or analyzed during the current study are available from the corresponding author on reasonable request.
