# Supplementary material for: A nomogram to predict ventricular thrombus in dilated cardiomyopathy patients
Source: J Thromb Thrombolysis. 2023 Jun 23;57(1):29–38. doi: 10.1007/s11239-023-02846-2 (PMC10830674; doi:10.1007/s11239-023-02846-2)
Supplement: Supplementary file 1 — Supplementary Material 1 [file 11239_2023_2846_MOESM1_ESM.docx]

**A Nomogram to Predict Ventricular Thrombus in Dilated Cardiomyopathy Patients**

**Table S1:** Criteria for ATRIA score

| **Risk factor** | **Points without prior stroke(points)** | **Points with prior stroke(points)** |
| --- | --- | --- |
| Age, years |  |  |
| >85 | 6 | 9 |
| 75-84 | 5 | 7 |
| 65-74 | 3 | 7 |
| <65 | 0 | 0 |
| Female sex | 1 | 1 |
| Diabetes mellitus | 1 | 1 |
| Congestive heart failure | 1 | 1 |
| Hypertension | 1 | 1 |
| Proteinuria | 1 | 1 |
| eGFR<45mL/min/1.73m2 or end-stage renal disease | 1 | 1 |

eGFR: estimated glomerular filtration rate

**Table S2:** Baseline characteristics of patients

| Variable | Non-VT group | VT group |
| --- | --- | --- |
| In-hospital new stroke | 32 (2.71%) | 7 (7.87%) |
| In-hospital mortality | 37 (3.14%) | 4 (4.49%)* |
| Days in hospital | 8.02±5.07 | 9.98± 5.61* |
| Distributions of VT |  | 89 |
| Left ventricular |  | 78 |
| left ventricular apical |  | 61 |
| middle left ventricle |  | 17 |
| Right ventricular |  | 7 |
| Biventricular |  | 4 |

VT, ventricular thrombus. *P<0.05

**Table S3:** Variable type conversion

| Variable | Cut-off value | Assignment | |
| --- | --- | --- | --- |
|  |  | 1 | 0 |
| Age, (years) | 58 | ≥58 | ＜58 |
| LVEF, (%) | 32 | ≤32 | ＞32 |
| AST, (U/L) | 48 | ≥48 | ＜48 |
| Creatinine, (ummol/L) | 83 | ≥83 | ＜83 |
| UA, (mmol/L) | 465 | ≥465 | ＜465 |
| NT-proBNP, (ng/mL) | 5820 | ≥5820 | ＜5820 |
| DD, (ng/mL) | 680 | ≥680 | ＜680 |

LVEF, left ventricular ejection fraction; UA, Uric Acid; NT-proBNP, N-terminal precursor B-type diuretic peptide; DD, D-dimer.

**Table S4:** Predictive probabilities of our nomogram versus other scores

| Index | Value | 95%CI | *P* value |
| --- | --- | --- | --- |
| NRI |  |  |  |
| vs CHA2DS2 | 1.38 | 1.23-1.53 | < 0.001 |
| vs CHA2DS2‑VASc | 1.41 | 1.26-1.55 | < 0.001 |
| vs ATRIA | 1.39 | 1.24-1.53 | < 0.001 |
| IDI |  |  |  |
| vs CHA2DS2 | 0.27 | 0.24-0.32 | < 0.001 |
| vs CHA2DS2‑VASc | 0.28 | 0.24-0.32 | < 0.001 |
| vs ATRIA | 0.27 | 0.23-0.31 | < 0.001 |

*CI, confidence interval;* NRI, net reclassification index; IDI, integrated discrimination index.


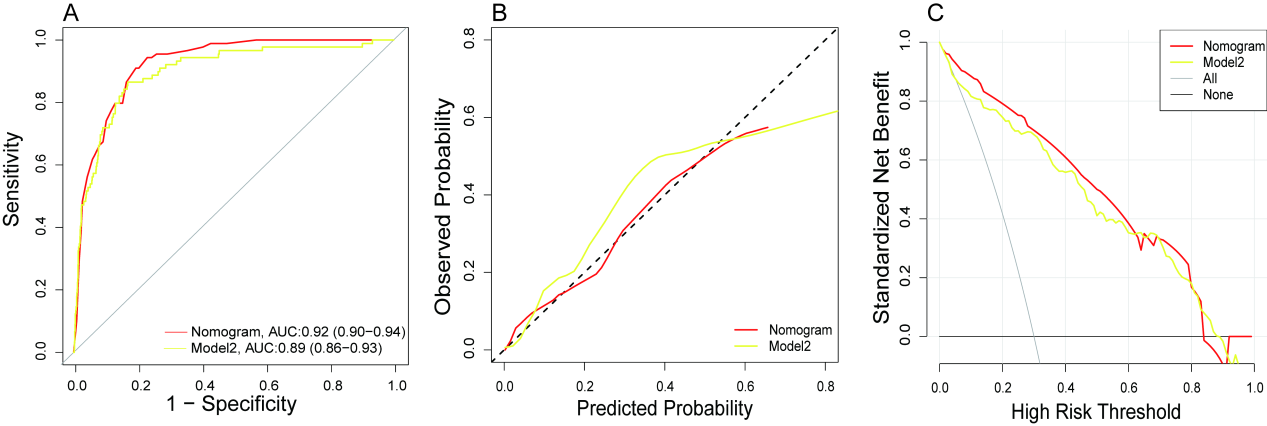


**Fig S1:** The comparisons between the model2 and nomogram. A, ROC curve comparing the model2 and nomogram; B, Calibration curve comparing the model2 and nomogram; C, Decision curve comparing the model2 and nomogram.
